# Assessment of Google Glass for Photographic Documentation in Veterinary Forensic Pathology: Usability Study

**DOI:** 10.2196/mhealth.9975

**Published:** 2018-09-21

**Authors:** Giuseppe Piegari, Valentina Iovane, Vincenzo Carletti, Rosario Fico, Alessandro Costagliola, Davide De Biase, Francesco Prisco, Orlando Paciello

**Affiliations:** 1 Department of Veterinary Medicine and Animal Production University of Naples Federico II Naples Italy; 2 Department of Pharmacy University of Salerno Fisciano Italy; 3 Department of Information and Electrical Engineering and Applied Mathematics University of Salerno Fisciano Italy; 4 Istituto Zooprofilattico Sperimentale delle Regioni Lazio e Toscana National Center for the Forensic Veterinary Medicine Grosseto Italy

**Keywords:** Google Glass, necropsy, pictures, documentation, veterinary forensic pathology, mobile phone

## Abstract

**Background:**

Google Glass is a head-mounted device designed in the shape of a pair of eyeglasses equipped with a 5.0-megapixel integrated camera and capable of taking pictures with simple voice commands.

**Objective:**

The objective of our study was to determine whether Google Glass is fit for veterinary forensic pathology purposes.

**Methods:**

A total of 44 forensic necropsies of 2 different species (22 dogs and 22 cats) were performed by 2 pathologists; each pathologist conducted 11 necropsies of each species and, for each photographic acquisition, the images were taken with a Google Glass device and a Nikon D3200 digital single-lens reflex (DSLR) camera. The pictures were collected, divided into 3 groups (based on the external appearance of the animal, organs, and anatomical details), and evaluated by 5 forensic pathologists using a 5-point score system. The parameters assessed were overall color settings, region of interest, sharpness, and brightness. To evaluate the difference in mean duration between necropsies conduced with Google Glass and DSLR camera and to assess the battery consumption of the devices, an additional number of 16 necropsies were performed by the 2 pathologists. In these cases, Google Glass was used for photographic reports in 8 cases (4 dogs and 4 cats) and a Nikon D3200 reflex camera in the other 8 cases. Statistical evaluations were performed to assess the differences in ratings between the quality of the images taken with both devices.

**Results:**

The images taken with Google Glass received significantly lower ratings than those acquired with reflex camera for all 4 assessed parameters (*P*<.001). In particular, for the pictures of Groups A and B taken with Google Glass, the sum of frequency of ratings 5 (very good) and 4 (good) was between 50% and 77% for all 4 assessed parameters. The lowest ratings were observed for the pictures of Group C, with a sum of frequency of ratings 5 and 4 of 21.1% (342/1602) for region of interest, 26% (421/1602) for sharpness, 35.5% (575/1602) for overall color settings, and 61.4% (995/1602) for brightness. Furthermore, we found a significant reduction in the mean execution time for necropsy conduced with the Google Glass with respect to the reflex group (*P*<.001). However, Google Glass drained the battery very quickly.

**Conclusions:**

These findings suggest that Google Glass is usable in veterinary forensic pathology. In particular, the image quality of Groups A and B seemed adequate for forensic photographic documentation purposes, although the quality was lower than that with the reflex camera. However, in this step of development, the high frequency of poor ratings observed for the pictures of Group C suggest that the device is not suitable for taking pictures of small anatomical details or close-ups of the injuries.

## Introduction

### Background

Google Glass is a device that was released for the first time as Google Glass explorer edition in 2013. It is a head-mounted device designed in the shape of a pair of eyeglasses with a 5.0-megapixel integrated camera; wireless connection; and the ability to take pictures, record a video, and call people with simple voice commands or manually by touching the frame. A small prism placed on the right side of the device allows a display of information to the user [1-3]. As a whole, the multitasking capabilities of the device provide users a comfortable and multifunctional virtual experience. Although these advantages have not fully met the needs of private consumers, its voice control, wireless transmission capabilities, integrated camera, and app customization have attracted the interest of commercial industries and professionals from various fields, including the health care [1,2]. In human medicine, Google Glass has been tested in many nonsurgical fields such as on-demand data visualization and real-time analysis [4], clinical simulations [5], management of diabetes [6], and pediatric cardiopulmonary resuscitation [7]. Furthermore, in neuropsychiatry, the usability and acceptability of Google Glass has been tested in children with autism spectrum disorder [8]. Similarly, in surgical settings, the multitasking capabilities of the device have allowed Google Glass to be tested in many surgical subfields such as cardiac surgery [9], neurosurgery [10], orthopedics [11], general surgery [12], and plastic surgery [13]. In these studies, Google Glass has been used as a tool to monitor vital signs, as an education instrument, and for telemonitoring and audiovisual recording. In human forensic pathology, Google Glass has been tested as a hands-free image acquisition device to document autopsies and postmortem examinations [14]. However, to the best of our knowledge, despite the several publications in human medicine, no empirical evidence for using Google Glass in veterinary medicine setting is currently known. The aim of this study was to determine the suitability of Google Glass in veterinary forensics pathology by assessing (1) the difference in mean duration between necropsies conduced with Google Glass and a digital single-lens reflex (DSLR) camera (Nikon D3200, lens AF-S DX Nikon 18-55 mm f/3.5-5.6G VR), (2) the battery consumption during the necropsies, (3) the usability aspects, and (4) the quality of the photographic documentation of the Google Glass compared with a DSLR camera.

### Veterinary Forensic Pathology

Over the last years, forensic necropsies in veterinary medicine have rapidly increased due to the increasing demand for investigations of crimes against animals. For these reasons, the subfield of veterinary forensic pathology has emerged as a distinct discipline, essentially based on a transverse, multiorgan approach that includes necropsy, histological examination, immunohistochemistry, and collateral examinations such as laboratory analysis and diagnostic imaging to resolve obscure fatalities [15-17]. The range of interests of veterinary forensic pathology is very broad and includes unlawful killing and animal abuse, diagnosis of drowning, nonaccidental injuries, violation of wildlife laws, malpractice or disciplinary procedures, and support to human forensic pathology [18-25]. Although there is no rigid scheme for veterinary forensic necropsies, the Italian Group of Veterinary Forensic Pathologists standardized a procedure for forensic autopsy cases, which is a useful guide that should be followed but, at present, is nonbinding. On the basis of this protocol, the forensic necropsy starts with victim’s identification and thanatological examination of the cadaver. Subsequently, a systematic evaluation of the external appearance of the animal is performed (outer necropsy). For this purpose, the head, mouth, mucous membranes, thorax, abdomen, perianal region, outer genitalia, hair coat, tail, as well as the front and hind limbs are surveyed. After that, the inner necropsy can start. During this step, a full skinning of the animal, inspection of the muscles and subcutis, as well as opening of all body cavities (skull, chest, abdomen, and pelvis) must be performed. Finally, all organs must be examined and dissected.

### Image Acquisition in Veterinary Forensic Pathology

Photography is an important component of documentation in forensic pathology [17,26,27]. A correct and complete photographic documentation is also expressly required by the guidelines for forensic veterinary autopsies issued by the Italian Group of Forensic Veterinary Pathology. During the forensic necropsies, photography is important to document both the presence (positive photograph) and absence (negative photograph) of injuries [28]; the main aim is the acquisition of images useful for legal purposes. Since necropsy is an unrepeatable procedure, the forensic photographic documentation should not only be accurate and detailed but also produce a minimal delay in the execution time of the necropsy [26]. Many DSLR cameras and mobile phones with photographic capabilities can be used for this purpose. However, these devices need to be used by qualified personnel with knowledge of photography and basics of veterinary forensic pathology in order to take clear and understandable pictures and minimize distortion and misleading information [14,26,29]. Usually, this assignment is delegated to veterinary forensic pathologists themselves because there are not professional figures suited to this purpose. Furthermore, during autopsies, pathologists are forced to a continuous replacement of gloves so as to use cameras for documentary purposes. These limitations result in an excessive workload for the pathologists, with consequent lengthening of the time required to perform the necropsy. In this context, it is easy to imagine the advantage of having a device that allows taking hands-free pictures.

## Methods

### Forensic Necropsy Protocol and Image Acquisition

A total of 44 necropsies of 2 different species (22 dogs, 22 cats; dogs: medium-sized, age range 6-9 years, mean age 7.31 [SD 1.04] years; cats: age range 6-9 years, mean age 7.00 [SD 0.92] years) were performed by 2 pathologists (FP and GP) with training in forensic medicine. All necropsies were performed in the necropsy room of the Department of Veterinary Medicine and Animal Productions at the University of Naples Federico II, Naples, Italy, following a standard necropsy protocol summarized in Textbox 1.

**Forensic autopsy protocol.**

1. Victim identification procedures
2. Evaluation of thanatological aspects and estimation of the time elapsed since death
3. External examination of the body (state of nutrition, mucous membranes, body orifices, general conformation, superficial lesions, hair coat, external parasites, and teeth)
4. Skinning with evaluation of subcutis and muscles
5. Opening and evaluation of body cavities (skull, thorax, abdomen, and pelvis)
6. Extraction and general macroscopic evaluation of organs
7. Dissection of all organs
8. Specific evaluation of wounds or injuries
9. Complete photographic documentation of external appearance of the animals, body cavities, organs, and injuries

Each pathologist conducted 11 necropsies of both species. For each photographic acquisition, the images were taken using two different devices: Google Glass and a Nikon D3200 DSLR camera. During the external inspection of the body, in accordance with the guidelines for forensic veterinary autopsies, pathologists were asked to take pictures of the external appearance of the animals from many different angles. Furthermore, during the necropsies, pictures of organs before and after extraction and any other detail useful for documentation purposes was acquired. All images were acquired under standard lighting conditions and without using the internal flash of the camera. In addition, a standard background (blue table of 90 × 70 cm) was used to acquire pictures of organs and small anatomical details. Finally, during image acquisition, a photomacrographic scale (American Board of Forensic Odontology No. 2 Standard Reference Scale) placed near the injuries was used to provide a geometrical reference in the forensic photographic documentation of the evidences.

### Evaluation of the Time of Necropsy and Battery Performance

To evaluate the differences in time of necropsy between autopsies conduced with Google Glass and DSLR camera and the battery performance of the devices, an additional number of 16 necropsies of 2 different species (8 cats and 8 dogs; dogs: medium-large dogs, age range 8-10 years, mean age 8.75 [SD 0.88] years; cats: age range 7-9 years, mean age 8.0 [SD 1.19] years) were performed by the 2 pathologists (FP and GP). In these cases, each pathologist conducted 4 necropsies of each species, with half of them conducted using Google Glass and another half using the Nikon D3200 DSLR camera. For each postmortem examination, we measured the time required to perform the necropsy using the stopwatch functionality available on a smartphone iPhone 6s Plus. To standardize the measurement, for all 16 forensic necropsies, the timer started at the first photographic acquisition and ended when the pathologist declared that he had acquired all pictures useful for the documentation purpose. Furthermore, each forensic examination began with devices (DSLR camera and Google Glass) charged to 100%, and at the end of each necropsy, the remaining battery power was noted.

### Usability Aspect

At the end of each necropsy performed with the Google Glass, pathologists were interviewed to acquire information about the user experience. The questions were designed to obtain information about the usability aspects, general experiences, and the main positive and negative features of the device.

### Google Glass

**Figure 1:**
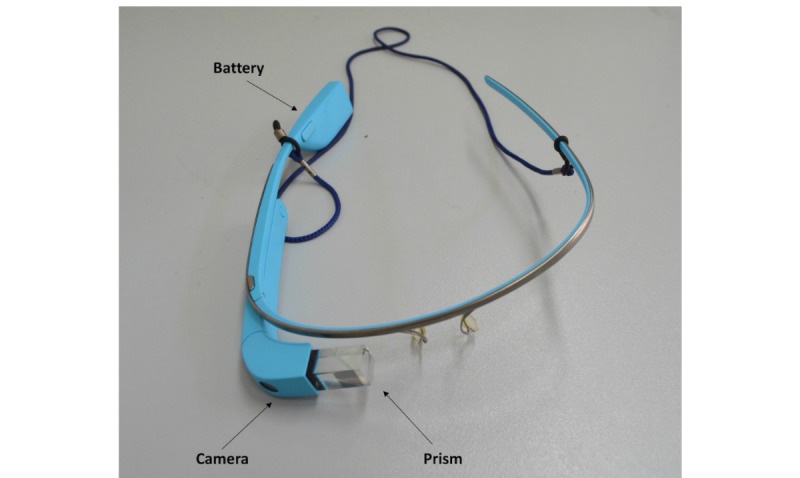
Google Glass device.

The device—a Google Glass explorer edition—available during our study, ran on Android 4.4.2. specifications of the available developer explorer unit that included Texas Instrument OMAP 4430 SoC 1.2 GHz Dual core (ARMv7) processor, 2 GB of RAM and 12 GB of usable storage space, a 640 × 360 display, 802.11b/g Wi-Fi, Bluetooth, and a 5-megapixel camera [3,14]. It also had a 3-axis gyroscope, a 3-axis accelerometer, a 3-axis magnetometer, ambient light sensor, proximity sensor, bone conduction audio transducer, and 2 omnidirectional microphones (Figure 1).

### Software Setup

For image acquisition, we used the preinstalled camera app for two reasons. First, we did not know whether the use of an app other than the preinstalled one would increase the battery consumption or decrease the photographic quality. Second, the voice commands and gestures performed on running the preinstalled app were easy to perform, intuitive, and precise; thus, we were not inclined to use an accessory app. However, to properly use the Google Glass camera during the necropsies, both pathologists followed a training course that lasted approximately 15 minutes. At the end of it, the pathologists declared to be able to use the devise correctly. The voice commands used during our study were as follows: “show viewfinder,” to frame the anatomical reason of interest correctly, and “take a picture,” to acquire the images. All accepted images were stored in a folder on the device until the pictures were transmitted via USB to an iOS-based laptop (MacBook Air 13").

### Digital Quality Image Assessment

A total of 5 forensic pathologists (AC, OP, RF, DDB, and VI; 4 males, 1 female; age range 33-58 years, mean age 42.60 [SD 9.37] years) with a mean work experience of 19 (SD 7.41) years in the field of veterinary pathology, both diagnostic and teaching, were selected to evaluate the quality of the images taken with both devices. To avoid compromising the evaluation of the images by the memories of the necropsies, the pathologists selected for the evaluation of the images were different from those who physically performed them. Furthermore, before the beginning of the evaluation, the pictures were divided into 3 groups: Group A, pictures of the external appearance of the animals; Group B, pictures of the organs; and Group C, pictures of small anatomical details or close-ups of the injuries. Each group included images taken with both devices. However, the device used for the acquisition was known (DSLR camera or Glass) only to the coordinators of the study. All 5 pathologists separately evaluated each group. In addition, all pictures were presented on a same computer (MacBook Air 13") with fixed display settings and similar environmental lighting conditions to avoid differences of evaluation due to external variables. The pathologists gave their opinions individually about the quality of the images analyzing 4 parameters: (1) overall color setting; (2) sharpness; (3) region of interest; and (4) brightness. Each one of these 4 parameters was separately evaluated on a 5-point score system according to Albrecht et al [14] (Table 1).

### Statistical Analysis

Statistics were computed using SPSS Version 22.0 (IBM Corporation, 2014, Armonk, NY, United States). Student’s *t* test was used to evaluate the difference in the mean duration between the necropsies conducted with Google Glass and the DSLR camera. The descriptive statistics for the ratings consisted of the tabulation of the frequency and percentages of scale items for each group for each item per device. To evaluate the differences between the ratings obtained for each group for both devices, we calculated an unpaired rank sum test (2-sided Mann-Whitney *U* test, with Cronbach alpha=.05) [14,30]. The same test was used to detect differences among the groups of the same device. To evaluate interrater reliability, we calculated intraclass correlation coefficients (ICCs) for the 5 pathologists for the items region of interest, sharpness, brightness, and color discrimination [31].

**Table 1:** Scoring system for image quality.

Parameter	Very poor	Poor	Average	Good	Very good
Sharpness	1	2	3	4	5
Overall color settings	1	2	3	4	5
Region of interest	1	2	3	4	5
Brightness	1	2	3	4	5

## Results

### Digital Image Quality Assessment

During the 44 forensic autopsies, the pathologists took 985 pictures with Google Glass and 985 pictures with the D3200 DSLR camera (504 photos of dogs and 481 photos of cats with each device). Each picture was evaluated by 5 pathologists, resulting in 4925 single evaluations each for Google Glass and the DSLR camera. Table 2 summarizes the results of the absolute frequencies and percentages of the ratings obtained per group for both devices for each of the 4 assessed parameters.

The ratings of the images taken with Google Glass during necropsies were significantly lower than those of the images acquired with the DSLR camera (Table 3). In particular, considering the percentage values, most ratings of the images taken with DSLR camera were high (good or very good) for all 4 parameters assessed. In contrast, for the images of Group A taken with Google Glass, the sum of frequency of ratings 5 (very good) and 4 (good) was 77.3% (1390/1800), 66.4% (1195/1800), 70.4% (1268/1800), and 71.7% (1290/1800) for region of interest, sharpness, brightness, and overall color settings, respectively. Furthermore, the images of Group B taken with Google Glass received a sum of frequency of ratings 5 and 4 of 54.7% (823/1505), 55.7% (838/1505), 65.8% (990/1505), and 54.0% (813/1505) for region of interest, sharpness, brightness, and overall color settings, respectively.

**Table 2:** Frequencies and percentages of evaluations given by 5 pathologists for images taken during forensic necropsies with Google Glass and Nikon D3200 reflex camera stratified for each group.

Parameters assessed and score^a^		Group A, n (%)				Group B, n (%)				Group C, n (%)		
		Glass (n=1800)	DSLR^b^ camera (n=1800)			Glass (n=1505)		DSLR camera (n=1505)		Glass (n=1602)		DSLR camera (n=1602)
**Region of interest**												
	5	426 (23.7)		933 (51.8)	173 (11.5)		522 (34.7)		55 (3.4)		1027 (63.4)	
	4	964 (53.6)		803 (44.6)	650 (43.2)		898 (59.7)		287 (17.7)		519 (32.0)	
	3	393 (21.8)		64 (3.6)	622 (41.3)		85 (5.6)		827(51.1)		74 (4.6)	
	2	17 (0.9)		0 (0)	60 (4)		0 (0)		451 (27.8)		0 (0)	
	1	0 (0)		0 (0)	0 (0)		0 (0)		0 (0)		0 (0)	
**Sharpness**												
	5	353 (19.6)		1005 (55.8)	37 (2.5)		434 (28.8)		125 (7.7)		762 (47)	
	4	842 (46.8)		708 (39.4)	801 (53.2)		929 (61.7)		296(18.3)		718 (44.4)	
	3	580 (32.2)		87 (4.8)	597 (39.7)		142 (9.4)		735 (45.4)		140 (8.6)	
	2	25 (1.4)		0 (0)	70 (4.6)		0 (0)		464 (28.6)		0 (0)	
	1	0 (0)		0 (0)	0 (0)		0 (0)		0 (0)		0 (0)	
**Overall color settings**												
	5	268 (14.9)		912 (50.7)	100 (6.6)		852 (56.6)		2 (0.1)		813 (50.2)	
	4	1022 (56.8)		783 (43.5)	713 (47.4)		619 (41.1)		573 (35.4)		742 (45.8)	
	3	507 (28.1)		105 (5.8)	582 (38.7)		34 (2.3)		770 (47.5)		65 (4.0)	
	2	3 (0.2)		0 (0)	110 (7.3)		0 (0)		275 (17)		0 (0)	
	1	0 (0)		0 (0)	0 (0)		0 (0)		0 (0)		0 (0)	
**Brightness**												
	5	285 (15.8)		997 (55.4)	204 (13.6)		680 (45.2)		125 (7.7)		880 (54.3)	
	4	983(54.6)		688 (38.2)	786(52.2)		806(53.6)		870 (53.7)		645 (39.8)	
	3	482 (26.8)		115 (6.4)	494 (32.8)		19 (1.3)		513 (31.7)		95 (5.9)	
	2	50 (2.8)		0 (0)	21 (1.4)		0 (0)		112 (6.9)		0 (0)	
	1	0 (0)		0 (0)	0 (0)		0 (0)		0 (0)		0 (0)	

^a^Parameters were assessed on a scale of 1-5 (1, very poor; 2, poor; 3, average; 4, good, 5, very good).

^b^DSLR: digital single-lens reflex.

**Table 3:** Unpaired rank sum, 2-sided Mann-Whitney *U* (Cronbach alpha=.05) for ratings of images taken with Google Glass and Nikon D3200 reflex camera for each group for each of the 4 assessed parameters.

Assessed parameters	Group A			Group B			Group C	
	z score	*P* value	z score		*P* value	z score		*P* value
Region of interest	−20.211	<.001	−26.771		<.001	−44.398		<.001
Sharpness	−26.468	<.001	−29.412		<.001	−37.705		<.001
Overall color settings	−25.177	<.001	−38.475		<.001	−40.931		<.001
Brightness	−26.786	<.001	−28.283		<.001	−31.452		<.001

The lowest ratings were observed in the pictures of Group C taken with Google Glass, with a sum of frequency of ratings 5 and 4 of 21.1% (342/1602) for region of interest, 26% (421/1602) for sharpness, 35.5% (575/1602) for overall color settings, and 61.4% (995/1602) for brightness. In this group, the differences between devices were particularly noticeable for region of interest, overall color settings, and sharpness. With regard to region of interest, the sum of frequency of ratings 2 and 3 amounted to 4.6% (74/1602) for images acquired using the DSLR camera versus 78.9% (1278/1602) for those acquired using Google Glass. Similarly, for overall color settings, this sum was 4% (65/1602) for images acquired using the DSLR camera versus 64.5% (1045/1602) for those acquired using Google Glass. Finally, for sharpness, the sum was 8.6% (140/1602) for images taken with DSLR versus 74% (1199/1602) for those taken with Google Glass. Furthermore, with regard to the pictures taken with Google Glass, statistical differences were observed in the distribution of ratings between Group A and Group B and between Group C versus B and A for all 4 assessed parameters (Table 4).

### Evaluation of Battery Performance and Time of Necropsy

Of the 16 necropsies conducted with Google Glass (8 necropsies) and DSLR camera (8 necropsies), we observed a reduction in the time of necropsy with Google Glass compared with that with the DSLR camera group. The mean duration of a single postmortem examination in the DSLR camera group was 126.38 (SD 3.46) minutes for the dogs group and 68.90 (SD 2.30) minutes for the cats group, whereas for the Google Glass, the mean duration was 111.11 (SD 3.29) minutes for dogs group and 55.5 (SD 2.06) for cats group. The differences were significant (*P*<.001). Furthermore, at the end of the necropsies conducted with Google Glass, the average percentage of battery power was 47% and 60% for the dogs and cats group, respectively (Table 5). For the DSLR camera, it was not possible to monitor the battery level because the display showed an icon with a crude scale in the unit of 33% and not the battery percentage. In any case, a significant battery consumption was not detected.

### Usability

**Figure 2:**
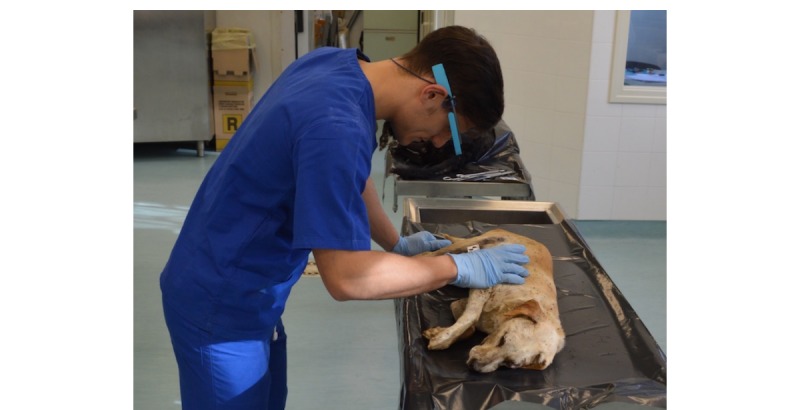
The forensic pathologist wears Google Glass and takes pictures of small anatomical details in a hands-free manner.

Based on interviews conducted at the end of the postmortem examination, we obtained subjective assessments about the user experience of Google Glass. As a positive aspect, the voice control was reported as useful, particularly in cases where both hands were occupied. The use of voice control led to increased saving of rubber gloves because the pathologists were not forced to take them off whenever they needed to take some pictures. In addition, the pathologists agreed about the ergonomics of the device and its lightness, which makes it comfortable to wear. As negative aspects, they reported the short battery life and difficulty to capture the desired regions of interest, especially for the close-ups. During use, the device was placed on the head and there was no zoom function available. For these reasons, the pathologists were forced to place themselves too close to the dissection table to be able to take pictures of small anatomical details or close-ups of the injuries correctly (Figure 2).

### Interrater Reliability

The interrater reliability was high. The ICC for the ratings obtained based on the forensic necropsy pictures indicated a good positive relationship for overall color settings (0.815, 95% CI 0.771-0.853; *P*<.001), region of interest (0.787, 95% CI 0.750-0.819; *P*<.001), sharpness (0.711, 95% CI 0.632-0.775, *P*<.001), and brightness (0.822, 95% CI 0.777-0.860; *P*<.001).

**Table 4:** Unpaired rank sum, 2-sided Mann-Whitney *U* (Cronbach alpha=.05) for ratings of the images of the 3 groups taken with Google Glass.

Assessed parameters	Group A versus B		Group B versus C		Group C versus A	
	z score	*P* value	z score	*P* value	z score	*P* value
Region of interest	−14.577	<.001	−22.603	<.001	−34.101	<.001
Sharpness	−11.396	<.001	−18.256	<.001	−26.095	<.001
Overall color settings	−12.740	<.001	−12.515	<.001	−25.412	<.001
Brightness	−2.737	.006	−5.052	<.001	−8.005	<.001

**Table 5:** Descriptive statistics of loss of battery consumption during forensic necropsy stratified by the device used to acquire the images: a Nikon D3200 reflex camera and the Google Glass device.

Device	Mean (SD)
Digital single-lens reflex camera	N/A^a^
Glass (dogs group)	47 (2.6)
Glass (cats group)	60 (2.9)

^a^N/A: not applicable.

## Discussion

### Principal Findings

Potentially disruptive technologies such as Google Glass create excitement for the possible applications that they can have in health care; however, the use of new tools should be thoroughly evaluated and validated before applying them in the medical or biomedical fields. Google Glass has been tested in many medical fields such as clinical simulations [5], surgery [9-13], and neuropsychiatry [8], but to the authors’ knowledge, this is the first study to assess the potential of Google Glass in veterinary medicine. Both devices tested in our study achieved the set goals and both allowed a complete photographic documentation. However, differences in efficiency between the two devices were observed. Our study showed a reduction in the necropsy time in forensic examinations conducted with Google Glass compared with those conducted with the Nikon D3200 DSLR camera; this was because Google Glass allowed hands-free operation, avoiding the continuous replacement of gloves that is necessary during necropsies performed with a reflex camera. Although glove saving was not a parameter directly evaluated in this study, this aspect was highlighted by both pathologists during the interviews conducted at the end of the necropsies. In addition, we observed a rapid reduction in the battery life of the Google Glass. In our opinion, power consumption was not a limiting factor because at the end of the necropsies, the average battery percentage was 47% and 60% for the dogs and cats groups, respectively. However, problems could arise in the case of more autopsies being performed on the same day. In these cases, pauses to allow battery charging should be considered. The difference in average Google Glass power consumption observed between the groups could be due to the different mean duration of a single postmortem examination of the dogs group (111.11 minutes) compared with the lower duration of the cats group (55.5 minutes) and, consequently, the mean duration of Google Glass use. Regarding the DSLR camera, it was not possible to monitor the battery level because the display showed an icon with a crude scale in units of 33% and not the battery percentage. In any case, significant loss of battery power was not detected. This is understandable considering the high capacity of the batteries commonly used in the modern DSLR cameras. Finally, images taken with Google Glass received significantly lower ratings for all 4 assessed parameters than those taken with the DSLR reflex camera. Most ratings for the images taken with the DSLR camera were high (good or very good) for all 4 assessed parameters. In contrast, in the pictures of the Group A taken with Google Glass, the sum of the frequency of ratings 5 (very good) and 4 (good) was 77.3% (1390/1800), 66.4% (1195/1800), 70.4% (1268/1800), and 71.7% (1290/1800) for region of interest, sharpness, brightness, and overall color settings, respectively; furthermore, the images of Group B taken with Google Glass received a sum of the frequency of ratings 5 and 4 of 54.7% (823/1505), 55.7% (838/1505), 65.8% (990/1505), and 54.0% (813/1505), respectively. The lowest ratings were observed for the pictures of group C with a sum of the frequency of ratings 5 and 4 of 21.1% (342/1602) for region of interest, 26.0% (421/1602) for sharpness, 35.5% (575/1602) for overall color settings, and 61.4% (995/1602) for brightness. This finding could be explained considering the lower camera resolution of Google Glass (5-megapixel camera with a fixed focal length of 3 mm) compared with the DSLR camera (24 megapixel) [32]. Hashimoto et al [12], in a recent study, reported that the video quality of iPhone 5 was greater than that of Google Glass during human surgical telementoring sessions. In contrast, some eye surgeons have reported a good video quality of the Google Glass device during scleral buckling surgeries [33]. These findings suggest that the camera quality of Google Glass is evaluated differently depending on the medical field of application and, consequently, on the photographic and recording quality required. Specifically, in our study, the gradual reduction in good or very good ratings observed among Groups A, B, and C could reflect the progressive increase in photographic quality required for the evaluation of anatomical details compared with that required for external examination for the evaluation of the body or organs. Albrecht et al [14], in a study conducted to assess the quality of the photographic documentation of the Google Glass device in human forensic pathology, showed a lower picture quality of the images taken with Google Glass than of those taken with a DSLR camera. In particular, the authors found that the differences between the devices were particularly noticeable only for region of interest and sharpness, whereas brightness and overall color settings showed similar distributions of ratings, with results slightly in favor of the pictures taken with the DSLR camera. These results appear in apparent contradiction with those obtained in our study. However, in this previous study, the images were evaluated as a whole and without being divided by type (external appearance, organs, and anatomical details). Furthermore, in the same study, an external application was used for image acquisition and a lower number of pictures was evaluated. However, excluding the methodological differences, the different results obtained in this study could be explained considering the different size of the organs of pet animals compared with human anatomy. These differences suggest a greater difficulty in the evaluation of images of organs and injuries of pet animals than that of humans. Similarly, for the external examination of the body, the difference in the size of animals compared with human anatomy and the presence of hair, common in all species of veterinary interest but absent in humans, makes the qualitative camera differences between devices more evident in animals than in humans. In addition, in our study, regarding the images taken with Google Glass, statistical analysis showed differences between the groups for all 4 assessed parameters. However, the highest frequency of rating 2 (poor) was observed for the pictures of Group C. The lower ratings observed for region of interest in Group C compared with those in Groups A and B was the most important aspect, and it could be explained considering that the Google Glass device had a wide-angle lens but not a zoom function [12,32]. During the forensic necropsy, the field of view of the Google Glass camera was too large, which forced the pathologists to place themselves too close to the dissection table to acquire pictures of small anatomical details. This led to the acquisition of poorly framed images that were unsuitable for forensic documentary purposes. Similarly, Moshtaghi et al [34], in a study conducted to assess the feasibility of using Google Glass during otorhinolaryngological procedures, found that the image quality was inadequate for viewing small and deep-seated anatomical structures. However, in our opinion, although this aspect has already been observed in human medicine, it could be even more relevant in pet animals than humans because of the differences in size between these species.

### Limitations

A few limitations of the study should be noted. First, we were able to test the device with only 2 pathologists. However, this allowed a more accurate assessment of the necropsy execution times. In our opinion, a greater number of pathologists would have determined a high variability in the time of necropsy due to the different levels of manual dexterity of each pathologist. Second, for the evaluation of the images, this study was based on subjective opinions of raters and not on objective and reproducible parameters. However, to reduce this limitation, a high number of highly qualified pathologists was selected for the evaluation of the images. In addition, the ICCs were evaluated for the 5 raters for the items region of interest, sharpness, brightness, and color discrimination. Furthermore, this study was conducted only on cats and dogs. We decided to test the device on these animals because although diagnostic necropsies are commonly performed on a broad range of animals, at present, they are the main species of forensic interest in the veterinary field [35,36]. However, this limitation makes these findings nonreproducible on other species of veterinary interest such as mice, rats, rabbits, or zoo and farm animals. Finally, the joint evaluation of the acquired images from 2 different species could be a further limitation of this study. However, considering the similar morphology and size of these animals (medium-sized dogs vs adult cats), we do not believe this to be a limitation.

### Conclusions

These findings suggest that Google Glass is usable in the veterinary forensic pathology of pet animals, but its image quality is lower than that of a reflex camera. In particular, the image quality of Groups A and B seemed adequate for forensic photographic documentation purposes. However, in this step of development, the high frequency of poor ratings observed for the pictures of Group C suggest that the device is not suitable for taking pictures of small anatomical details or close-ups of the injuries. In our opinion, the combined use of the two devices, reflex camera for capturing images of small anatomical details and Google Glass for capturing images of the external appearance of the animals and organs, could reduce the execution times of the necropsy, lead to considerable saving of gloves, and allow acquisition of pictures useful for forensic documentation purposes. However, further studies will be needed to evaluate the application of this device to other species of veterinary interest such as wildlife or farm animals. In some of these species, the greater volume of the organs than that in pet animals could make the qualitative differences between Google Glass and the reflex camera less evident but, above all, could make the absence of the photographic zoom in Google Glass less limiting.

## Abbreviations

DSLR: digital single-lens reflex
ICC: intraclass correlation coefficient
